# Influenza-Related Encephalopathy in Children: Epidemiology and Literature Review from a Tertiary Hospital in Northern Italy (Winter 2023–2024)

**DOI:** 10.3390/pathogens14060551

**Published:** 2025-06-01

**Authors:** Raffaele Vitale, Marco Denina, Laura Badiali, Matteo Sandei, Giulia Mazzetti, Carlotta Canavese, Aba Tocchet, Antonia Versace, Silvia Garazzino

**Affiliations:** 1 Infectious Diseases Unit, Department of Pediatrics, Regina Margherita Children’s Hospital, A.O.U. Città Della Salute e Della Scienza di Torino, 10126 Turin, Italysilvia.garazzino@unito.it (S.G.); 2 Department of Public Health and Pediatrics, Università Degli Studi di Torino, Piazza Polonia 94, 10126 Turin, Italymatteo.sandei@unito.it (M.S.);; 3 Child and Adolescent Neuropsychiatry Unit, Department of Sciences of Public Health and Pediatrics, A.O.U. Città Della Salute e Della Scienza di Torino, 10126 Turin, Italy; 4 Department of Pediatric Emergency, Regina Margherita Children’s Hospital, Città Della Salute e Della Scienza, 10126 Turin, Italy; aversace@cittadellasalute.to.it

**Keywords:** influenza, encephalopathy, encephalitis, children, necrotizing encephalitis

## Abstract

Introduction: While influenza-associated encephalopathy (IAE) in children remains a serious concern, recent evidence suggests a shift in its epidemiology, with a possible decline in incidence and severity over time. Methods: This retrospective review includes patients aged 0–18 admitted to a tertiary hospital in Northern Italy between November 2023 and February 2025. Inclusion criteria were a positive influenza test, influenza-like symptoms, and neurological involvement. Findings are interpreted alongside current literature. Results: Twenty-five unvaccinated children met criteria for IAE (11 in 2023/24; 14 in 2024/25). Neurological comorbidities were present in 40%. All patients had pathological EEGs. Lumbar puncture was performed in 40%, with abnormalities in 33%. Brain imaging was conducted in 56% of cases, revealing findings in 15%. All received oseltamivir; 60% were also treated with dexamethasone. Severe complications occurred in 16%, while 12% had persistent symptoms or required therapy at discharge. After adjusting for seasonal peak timing, no significant inter-seasonal difference was observed. Discussion: Although IAE continues to present serious risks, recent trends suggest a changing burden. The lack of vaccination among cases underscores the need for prevention. Study limitations include its single-center design and the absence of long-term follow-up. Broader prospective studies are needed.

## 1. Introduction

Influenza-associated encephalopathy (IAE) and encephalitis are rare but serious complications of seasonal influenza in children. Neurological manifestations—ranging from febrile seizures to acute necrotizing encephalopathy—have been consistently reported, especially in younger age groups [1,2,3]. However, the burden of IAE appears to have evolved significantly over the past decade.

A 2025 report by Fazal et al., based on CDC surveillance data, reviewed pediatric influenza-associated deaths from the 2010 to 2025 seasons and found that the proportion of deaths attributed to IAE peaked at 14% in 2011–2012 but dropped to 0% in 2024–2025, suggesting a substantial decline in the severity of neurological involvement over time [4]. These findings likely reflect a combination of increasing population immunity, earlier antiviral interventions, and viral evolution.

In Italy, national surveillance from 2017 to 2019 recorded 15 cases of IAE in children, equating to an incidence of 7 per million per season—consistent with our observed rate of 6.8 per million per season [5]. A 2024 multicenter study in France by Savagner et al. reported neurological symptoms in 10.3% of pediatric influenza hospitalizations, with febrile seizures being the most frequent. Remarkably, 93% of affected patients returned to their neurological baseline at discharge, and severe forms, such as acute necrotizing encephalopathy, were rare [1].

This changing landscape is further supported by studies from Asia. In Thailand, Jantarabenjakul et al. (2023) found neurological complications in 7.6% of pediatric influenza admissions, mainly febrile seizures and encephalopathy [6]. In China, Yang et al. (2024) observed a 16% rate of IAE in children, with prognosis closely linked to Glasgow Coma Scale score at admission and early oseltamivir administration [7]. Li et al. (2024) developed a predictive model identifying high fever, leukocytosis, and preexisting epilepsy as key risk factors for neurological complications [2].

These recent data contrast with findings from the 2009 H1N1 pandemic, a period during which IAE was disproportionately common and often severe. That era defined much of the early literature and shaped clinical awareness of influenza-related neurological syndromes. However, it is now evident that the epidemiology of IAE has shifted significantly, underscoring the need for updated clinical insight and risk assessment.

Current evidence suggests that IAE is driven primarily by systemic immune dysregulation—including cytokine storm and endothelial damage—rather than direct viral neuroinvasion. Antiviral therapy (e.g., oseltamivir) is routinely used, although its effect on neurological outcomes is still under investigation. Corticosteroids are sometimes administered in severe presentations, but their efficacy remains inconsistent across studies [7].

Despite mounting evidence that influenza vaccination reduces both IAE incidence and severity, pediatric coverage remains inadequate in many regions. In Italy, vaccination is recommended for children aged 6 months to 6 years but remains far below the EU target of 75% [5]. Strengthening public health efforts to raise vaccination uptake could prove essential in mitigating neurological complications in pediatric influenza.

This study aims to characterize the clinical features, management, and outcomes of children with IAE admitted to a tertiary pediatric center in Northern Italy over two recent influenza seasons (2023–2025), contextualized within the most current international data.

## 2. Materials and Methods

### 2.1. Study Design and Setting

This was a mixed-model, single-center study conducted at Regina Margherita Children’s Hospital in Turin, a tertiary inter-regional referral center for pediatric care in Northern Italy. The study spanned two consecutive influenza seasons: data for the 2023–2024 season were collected retrospectively, while the 2024–2025 season involved prospective patient enrollment.

### 2.2. Study Population

Children aged 0–14 years who were admitted with laboratory-confirmed influenza (type A or B) and new-onset neurological symptoms were included. Eligible patients were hospitalized in either the pediatric ward or the pediatric intensive care unit (PICU) during the study periods.

### 2.3. Diagnostic Methods

Influenza infection was confirmed by real-time multiplex PCR using either the BIOFIRE^®^ FILMARRAY^®^ Respiratory Panel (bioMérieux, Craponne, France) or the Xpert^®^ Xpress Flu/RSV assay (Cepheid, Sunnyvale, CA, USA). Both platforms are validated for the differentiation of influenza A and B viruses.

### 2.4. Ethical Considerations

This study was conducted in accordance with the principles of the Declaration of Helsinki, and written informed consent was obtained from all participants. The study was approved by the local ethical committee: “Comitato etico territoriale Interaziendale A.O.U Città della Salute e della Scienza di Torino of the Coordinating Center. Approval code: 0049894; approval date 5 May 2022”. For children old enough to understand, the study was explained in age-appropriate language before enrollment.

### 2.5. Statistical Analysis

Categorical variables were compared using the χ^2^ test or Fisher’s exact test, as appropriate. Continuous or non-normally distributed variables were analyzed using the Mann–Whitney U test. To compare the incidence of neurological complications between the two seasons, a log-rank test was performed, with statistical significance set at *p* < 0.01. Assumptions regarding the independence of observations and non-informative censoring were verified. All analyses were conducted using R software (version 4.1.2), employing the following packages: stats for core statistical functions, ggplot2 for data visualization, and survival, survfit, boot, and pracma for survival analysis and curve estimation.

## 3. Results

**Figure 1:**
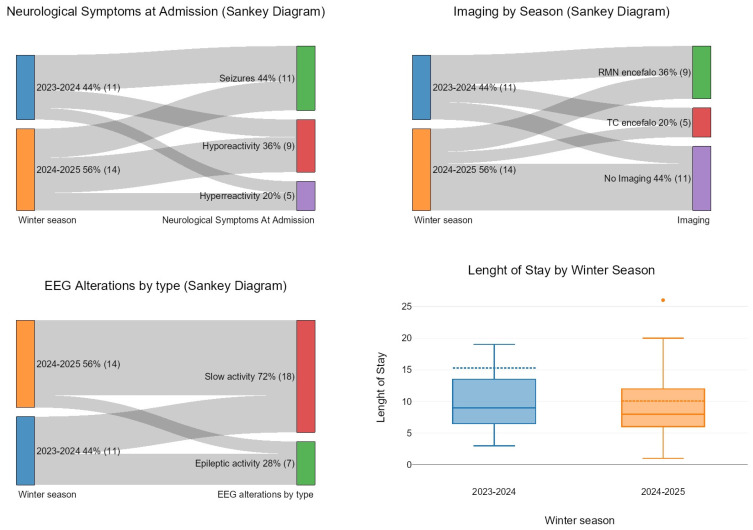
Diagram of remarkable population features.

Baseline characteristics of the study population are shown in Table 1 and Figure 1. A total of 25 patients were included, with 11 cases occurring in the winter of 2023/2024 and 14 cases in the 2024/2025 season. For composite variables such as “underlying neurological conditions”, we included cases of hypoxic–ischemic encephalopathy, brain tumors, one case of tuberous sclerosis, and known epilepsy at admission. Respiratory involvement was defined as any signs or symptoms of respiratory distress requiring supplementary oxygen or ventilation, as well as clinically or radiographically confirmed pneumonia. “Pathological cerebrospinal fluid (CSF)” was defined as any abnormalities detected via multiplex PCR, chemical, or cytological analysis. Brain MRI was performed following an initial CT scan, which had been conducted in an emergency setting before lumbar puncture. Dexamethasone was the only glucocorticoid used in our patients, administered at a dose of 0.1 mg/kg four times daily.

**Figure 2:**
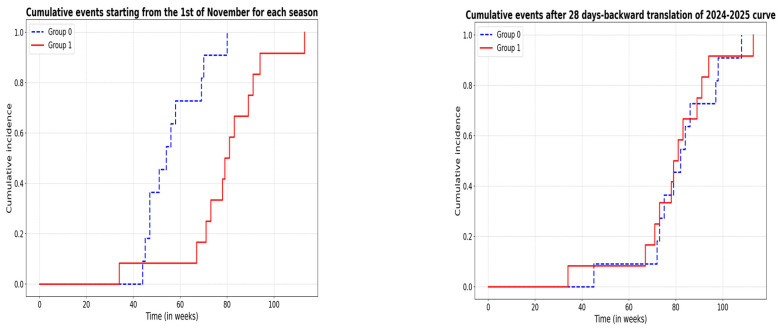
Cumulative incidence curves of influenza-related encephalopathy in two winters. To the left: uncorrected curve; to the right: curves corrected for peak-to-peak difference in incidence.

Before analyzing time to event differences among groups, we evaluated for gross discrepancies in Italian incidence curves: we calculated, for each season, the AUC from the incidence curve week by week [8], with AUC 220 for winter 2023–2024 and AUC 239 for winter 2024–2025, with an observed difference of 19. A bootstrap test with 10,000 resampling iterations showed a 95% CI: −340.15, 314.20, indicating that the AUC difference is statistically non-significant. However, we acknowledge that the incidence peaks are separated by 4 weeks, and we tried to compensate for that. First, we performed log-rank testing on a zero model not accounting for differences in the distribution of seasonal flu pattern in Italy (the starting point is the first of November of each year). There is a significant difference in survival between Winter 2023–2024 (Group 0) and Winter 2024–2025 (Group 1) (χ^2^ = 15.2, *p* = 0.0005). The second step was performing a log-rank test conducted on an “incidence-informed” model in which we performed an affine transformation (translation of one curve by a module of “28 days”, which is the peak-to-peak difference of the two distributions patterns). In this model, difference disappeared (χ^2^ = 0.7, *p* = 0.4) (Figure 2).

## 4. Discussion

This study investigated the neurological complications of influenza infection in children in Northern Italy during two winter seasons (2023/2024 and 2024/2025). Epidemiological data for these seasons were provided by the Italian National Health Institute [8]. The 2023 peak occurred earlier, in the 52nd week of the year (18.45/1000 vs. 10.54/1000 for the same week in 2024–2025, no age restriction), while the 2024–2025 peak occurred in the 4th week of January (9.9/1000 vs. 17.36/1000, no age restriction). During the 2023–2024 influenza season in Italy, the most prevalent serotype was A(H1N1)pdm09, comprising 94% of type A strains. Type B viruses accounted for 6%. Although data for the 2024–2025 season remain partial, type A continues to dominate (64%, with 66% H1N1 and 33% H3N2), while type B has risen to 23%. We could not serotype the subclass of the A group for all patients; however, based on national epidemiological trends, we postulate that H1N1 was the most frequent strain in both seasons. Viral co-infection was identified in three cases (two with rhinoenterovirus and one with respiratory syncytial virus), while no cases of bacterial co-infection were documented.

Historically, H1N1—especially during its early post-pandemic circulation (2009–2012)—has been linked to higher rates of neurological complications in children. However, the persistence of this association in recent years remains uncertain, as many contemporary studies do not consistently disaggregate neurological outcomes by viral subtype [1,2,3,4,5,6,7,8,9]. While our results align with prior literature associating H1N1 with influenza-associated encephalopathy (IAE), further subtype-specific epidemiological analyses are necessary to assess whether this relationship remains valid in the current era. By contrast, influenza A (H3N2) is more consistently linked with severe respiratory disease and higher mortality, possibly due to greater neuraminidase activity and distinct host interactions [1,10]. Notably, during a recent H3N2 outbreak in Shenzhen, a significant number of pediatric neurological complications were reported, including cases of encephalopathy, underscoring the neurovirulent potential of this subtype as well [11]. The cumulative incidence of influenza across the two seasons was similar (as indicated by non-significant differences in AUC curves), though the distribution over time differed—peaking approximately four weeks earlier in 2023. This temporal mismatch likely explains the unadjusted discrepancy in case counts. When this peak shift is corrected for, survival analysis no longer indicates a significant seasonal difference, suggesting that timing rather than strain severity may account for apparent disparities.

While foundational studies of IAE stemmed from the heightened concern during the 2009 H1N1 pandemic, more recent literature and surveillance data indicate that the association between H1N1 and neurological complications has persisted over time. A 2023 bibliometric analysis of influenza encephalopathy research from 2000 to 2022 identified a publication cluster focused on H1N1-linked encephalopathy during the 2009–2012 period, but also showed sustained scientific interest in this subtype well into the following decade, reflecting ongoing clinical relevance [12]. This continuity is further supported by recent epidemiological and hospital-based studies across different countries that continue to report H1N1 as the most frequently implicated strain in pediatric neurological complications [1,4,5,7].

Our study thus emphasizes the importance of continuously updating clinical understanding based on current strain behavior and host susceptibility profiles. Emerging data also highlight that influenza B, although less frequently implicated overall, may pose considerable risk to immunologically naïve pediatric populations during its cyclical circulation. This evolving pattern underscores the need to interpret strain-specific risks within the context of shifting immunity and viral dynamics [9,10].

While influenza A remains the most frequently implicated subtype in neurological complications, emerging data suggest that influenza B may also pose a substantial risk during its cyclical circulation, particularly in immunologically naïve pediatric populations [4,9,10]. The intermittent pattern of influenza B circulation—often with multi-year gaps between dominant seasons—can lead to a lack of population-level immunity in younger cohorts. This may predispose these children to more severe clinical outcomes, including neurological complications, in seasons where influenza B is more prevalent [8]. Although our dataset predominantly involved influenza A and did not allow for subtype-specific outcome comparisons, acknowledging the growing relevance of influenza B is critical for anticipating future pediatric disease burdens and informing vaccination strategies. Broader surveillance and more granular serotyping in future studies would be valuable to assess the true neurotropic potential of influenza B relative to A in children.

Single-case studies describe encephalopathy in adults [13], but children aged 0–5 years appear to be the most vulnerable age group, if not exclusively affected. The median age of our patients with neurological involvement was 3.69 (IQR 3.86) (drawing on patients aged 0–16 years), in line with that reported in the literature [1,6,7,14,15,16,17,18,19,20]. Preexisting neurological conditions are associated with both a higher risk of developing the disease and a more severe form [1,7,9,10,13] (20–40% in different publications [5,7,9,10,14,15], similar to our 40%). That was also confirmed by a recent May 2025 paper on 70,000 children with influenza [16]. None of the patients included in our cohort had a condition of primary or secondary immunodeficiency, including cancer, immunosuppressive therapy, or end-stage renal disease. Neither the patients nor their close relatives received vaccination for the current flu season. Vaccination has been strongly associated with reduced complications, including neurological ones. The 2024 study by Yang [6] and the 2024 alert from the Italian group (Bartolini et al.) [21] on pediatric influenza encephalitis, in which none of the patients were vaccinated, support this. The same study by Yang [7] showed that influenza vaccination could reduce hospitalizations in the PICU by 74%. Similar results [18] were reported by Frankl et al. (2022), who found that only 50% of total hospitalizations and 55.8% of hospitalizations with a pre-existing neurological diagnosis had been vaccinated, highlighting opportunities for improvement in both overall and targeted vaccination strategies. As mentioned, the influenza virus causes minimal, if any, direct damage to the central nervous system. The main mechanism of neurological involvement appears to be secondary to cytokine release as an inflammatory response to the viral infection, endothelial dysfunction, and a susceptible genetic background [22]. In our population, the main signs/symptoms were flu-like (rhinoconjunctivitis, reduced feeding, asthenia). Fever (body temperature > 38 °C) was reported in all cases. Upper respiratory symptoms were present in more than 90% of cases, while lower respiratory involvement was recorded in 20% of patients, equally distributed across the two seasons. These features are in line with what is reported in the literature. Regarding neurological involvement, we reported seizures (n = 11, 40%), severe hyporeactivity (n = 69, 36%), and agitation (n = 5, 20%). These proportions were consistent across the two seasons. Febrile seizures have been found to be the most common neurological complication in children hospitalized with influenza (66% of cases in the literature [1,7,16,17,18,19,20], 40% in our study). These manifestations—fever, febrile seizures, and signs of upper or lower respiratory tract infection—were also the typical presenting symptoms at the time of emergency department evaluation, prompting influenza testing and subsequent admission. In one study, febrile seizures accounted for 80.6% of influenza-associated neurological complications [20]. Altered mental status was present in approximately 55% of cases across different studies [1,19,22]. We observed one case of severe neurological involvement requiring intensive care, which was the only case of necrotizing encephalitis. This entity represents the severe end of the disease spectrum and carries a high mortality rate. In a 2024 Chinese study on 74 neurological influenza cases, it represented 11% [7]. Mortality in this condition ranges from 30% to 50%, according to studies [7,20,22,23]. No patient died in our study, but we recorded one case of severe necrotizing encephalitis. Other severe manifestations, such as ADEM and Guillain–Barré syndrome, were not encountered in our study. This is consistent with the low incidence of these conditions (less than 0.1 per 100,000) [24]; they are rare events, even with the logistical advantage of working at a tertiary pediatric center. The median time from flu-like symptoms to neurological onset was very short (2, IQR 2.25), consistent with what is reported in the literature [10,14,17,19]. EEG abnormalities were observed in every patient, with slow-wave patterns being the most frequent (82%). Epileptic activity occurred in seven patients (28%). Reported rates vary across studies and were as low as 49% in a recent 2024 French study [1] involving 131 patients with neurological influenza. Although not necessary for diagnosis, EEG alterations are helpful in addressing it, so a selection bias at our center cannot be excluded. Severe depressive patterns, such as “generalized slow waves” or “extreme delta brush”, observed in our most severe patient, are well known to be associated with necrotizing encephalitis or, at the very least, a worse prognosis [7,20]. A lumbar puncture was performed in 40% of cases, always after brain imaging, to reduce procedural complications and guide diagnosis. Imaging (MRI or CT) was performed in 14 patients (61% of cases), with pathological findings in only 2 patients, both of whom belonged to the 2023–2024 group. Brain CT was requested in four patients (75% of which were in the 2023/2024 winter group) when a fast scan was needed (severe disease, pneumonia). These patients were subsequently referred for MRI. Interestingly, CSF analysis showed pathological findings in only three patients (CSF was considered abnormal when there were more than 5 white blood cells per mm^3^ (excluding newborns), protein levels were elevated above 58 mg/dL, or there was a CSF-to-serum glucose ratio below 0.6, as these findings may indicate central nervous system infection or inflammation) [25]. Influenza PCR tests are not available at our center. The decision for both imaging and subsequent lumbar puncture was made on a case-by-case basis, depending on the severity of symptoms and the presence of a presumptive causative agent (in this case, the influenza virus). Brain CT or MRI scans were normal in all but one case (the most severe involvement). As the literature shows, alterations are not typical, and prognosis in influenza encephalopathy/encephalitis may correlate with these alterations [26]. MERS (mild encephalitis/encephalopathy with reversible splenial lesion, hyperintensity in the posterior matter) is correlated with a better outcome, while typical patterns of necrotizing encephalitis (such as swollen, T2 hyperintense basal ganglia) are associated with both the need for intensive care and death, as seen in the 2024 Italian study on six severe influenza encephalitis cases [21]. In every study, patients were treated with oseltamivir regardless of the time elapsed since the first symptoms appeared. The mean time from symptom onset to the first oseltamivir dose was 2 days, while the median duration of therapy was 5 days (IQR: 0; max value: 10 days in the acute necrotizing encephalitis case). In our investigation, oseltamivir was administered for neurological symptoms in every case. The median time from symptom onset to therapy initiation that is reported is 2–3 days [7,17,21,27]. Its direct efficacy has been claimed in single-case reports, but more studies are needed [23,28]. A 2021 Chinese study on 118 children with severe influenza showed a trend toward worse outcomes (including neurological) for those who started antiviral therapy more than 2 days after symptom onset [23]. The use of corticosteroids in treating infectious encephalitis is still debated, as their efficacy on both mortality and disability varies [1,14,18,28,29]. In influenza encephalopathy, these drugs are reserved for severe forms [6,14,18,28,29]. A 2024 Vietnamese study (n = 20, mean age 5 years) on the timing of administration failed to demonstrate better long-term outcomes if methylprednisolone was administered within 48 h of the onset of neurological symptoms [26]. Severe adverse effects of steroid therapy, such as hypercoagulability and overwhelming opportunistic infections, have been reported [2,5,20]. The percentage of patients treated with dexamethasone was 64% in our study, with dexamethasone as the steroid of choice and no difference in administration proportions between the seasons. Doses ranged from 0.4 to 0.6 mg/kg/day (none received methylprednisolone pulse therapy). The “longer” duration of dexamethasone therapy in the 2023 season is attributed to the outlier effect of one severe case (70 days of therapy). Methylprednisolone appears to be the most commonly used corticosteroid in the reviewed studies, often administered in pulse therapy or at variable dosages (1–2 mg/kg/day). Dexamethasone is mentioned as a low-dose alternative (0.2–0.3 mg/kg/day). The specific use and dosage depend on the hospital’s treatment protocol, the severity of the condition, and the patient’s response [1,18,28,29]. We will not elaborate further on this topic, as the duration of glucocorticoid therapy was tailored to the clinical response, so we cannot infer a causative effect on length of stay or residual disability at discharge. The 2023 cohort’s longer therapy was skewed by one necrotizing encephalitis patient requiring over 70 days of treatment. EEG normalization was required for discharge. New antiepileptic therapy at discharge, as an indicator of residual involvement, was prescribed in 13% of cases (three patients), including two from the 2023–2024 cohort. In addition to the necrotizing form, those patients had preexisting neurological conditions. One year later, those on antiepileptics had not experienced any seizures.

The necrotizing encephalitis patient had the worst one-year prognosis, characterized by severe psychomotor delay, persistent diffuse cerebral damage with epileptiform abnormalities, slowed nerve conduction (auditory, somatosensory, and optic pathways), and gastrostomy dependency. Upon discharge, he was sent to a rehabilitation center, where he stayed for two months. Every other patient was safely discharged home. No death was observed. In a 2021 study, Frankl et al. (2021) [18] reported 11% of cases with neurological complications, while in the 2024 study by Savagner et al. (2024) [1], 93% of patients had a favorable outcome at discharge, with 4.6% of patients not returning to their neurological baseline at discharge. Neurological complications included febrile and non-febrile seizures, status epilepticus, encephalopathy, meningitis/encephalitis, ataxia, cerebral edema, arterial ischemic stroke, and transverse myelitis. That was confirmed in other studies too [11,14,15,30]. To prevent neurological influenza complications, seasonal vaccination could be an essential tool in the public health armamentarium. Although it has been demonstrated that it reduces complications, including neurological ones, pediatric coverage is low in many countries [2,7,17,31,32]. In Italy, the influenza vaccine is provided free to individuals over 60 and specific at-risk categories. Since 2020, vaccination has also been recommended for all children aged 6 months to 6 years, but coverage remains below the European Union’s target of 75%. In 2023/2024, the vaccination coverage in the general population decreased to 18.9%, compared to 20.2% in 2022/2023 [31].

No patients in our two-year cohort were vaccinated for influenza, supporting the need for improving public health policies to overcome parents’ hesitancy and achieve a higher rate of flu vaccine coverage.

Similar results are observed in United States, where flu vaccination coverage for pediatric population (up to 17 years old people) in 2023–2024 was 55.4% (2% lower than the previous year and significantly lower than pre-pandemic 2019–2020 (63.7). The last time flu vaccination coverage among children was lower was in winter 2011–2012 (51.5%), and that is the season in which the highest incidence of influenza encephalitis was reported in the US [32].

Additional insights were gained by analyzing data on febrile convulsions in our broader pediatric population. Between November 2023 and April 2025, 175 children under 5 years of age were admitted to our hospital for febrile convulsions. Among them, 25 (14.3%) were diagnosed with influenza-associated encephalopathy, a proportion consistent with our main study cohort. Although our study did not directly compare influenza-infected versus non-influenza febrile children, these findings suggest that influenza may contribute to a substantial subset of neurological complications in this vulnerable age group. Moreover, none of the influenza-positive children had received vaccination. While we did not perform a formal case–control comparison, this observation supports prior literature indicating a potential protective effect of seasonal influenza vaccination against severe neurological complications. Future studies examining febrile seizure subgroups by viral etiology and vaccination status would help clarify these associations.

Limitations of our study include the fact that this was a single-center experience, albeit at a tertiary-level center. Serotyping of the influenza virus was not always possible. The ongoing influenza season prevents us from offering an opinion on the long-term prognosis of those with residual neurological symptoms at discharge.

## 5. Conclusions

Influenza-associated encephalopathy remains a serious complication of viral infection in children, though its epidemiology has evolved significantly since the 2009 H1N1 pandemic, when neurological involvement was markedly more frequent and severe. More recent data suggest a shift in both incidence and severity, with a trend toward lower complication rates, likely influenced by viral evolution, population immunity, and improvements in clinical management. Nevertheless, the continued occurrence of severe cases—particularly among unvaccinated and neurologically vulnerable children—underscores the need for ongoing surveillance and proactive prevention strategies. Seasonal influenza vaccination remains a critical tool in reducing the burden of neurological complications in pediatric populations.

## Figures and Tables

**Table 1:** Baseline features of the study population.

	All Patients	2023–2024 Winter Season	2024–2025 Winter Season	Statistics */**
*n* (%)	25 (100)	11 (44)	14 (56)	
Age at admission (in years), median (IQR)	3.69 (3.86)	5 (5.5)	4 (3.5)	** p.80
Sex (male/female): *n* /*n* (%/%)	14/11 (56/44)	6/4 (32/24)	8/6 (41.5/58.5)	* p.89
Neurological comorbidity § *n* (%)	10 (40)	3 (12)	7 (28)	* p.25
Influenza B type	4 (16)	0 (0)	4 (28.5)	* p.53
Underlying neurological conditions at admission:				* p.87
• Hyporeactivity *n* (%)	9 (36)	3 (12)	6 (24)	
• Hyperreactivity *n* (%)	5 (20)	2 (8)	3 (12)	
• Seizures n (%)	11 (40)	6 (24)	5 (20)	
Time (days) from flu-like to neurological symptoms, median (IQR)	1 (3)	1 (1)	2 (3.7)	** p.768
Respiratory distress/involvement ‡ n (%)	9 (36)	4 (16)	5 (20)	* p.648
Gastrointestinal symptoms *n* (%)	7 (28)	2 (8)	5 (20)	* p.332
C-Reactive protein mg/dl, median (IQR)	4.3 (16.3)	11 (21)	8.85 (11.4)	** p.312
White blood cells/mmc, median (IQR)	8000 (6815)	8120 (4255)	7420 (6515)	** p.384
Neutrophils/mmc, median (IQR)	4105 (5390)	4860 (4630)	2800 (4160)	** p.536
Lymphocytes/mmc, median (IQR)	1300 (1502)	1020 (1143)	1400 (2130)	** p.151
Lumbar tap *n* (%)	10 (40)	5 (20)	5 (20)	* p.69
Pathologic findings ⁋	3 (33)	3 (100)	0 (0)	
EEG anomalies at admission:	25 (100)			* p.177
• Slow patterns *n* (%)	18 (72)	6 (24)	12 (48)	
• Epileptic features *n* (%)	7 (28)	5 (20)	2 (8)	
Brain imaging *n* (%)	14 (56)	8 (72)	6 (43)	* p.144
Brain TC *n* (%)	4 (31)	3 (27)	1 (7)	* p.144
Only brain MRI *n* (%)	9 (36)	4 (36)	5 (36)	* p.144
Pathologic findings *n* (%)	2 (15)	2 (100)	0 (0)	* p.5
Acyclovir *n* (%)	11 (44)	6 (24)	5 (20)	* p.435
Acyclovir duration (days), median (IQR)	6 (5)	3.5 (6)	7 (1)	** p.792
Oseltamivir duration (days), median (IQR)	5 (0)	5 (0)	5 (0)	** p.525
Glucocorticoids *n* (%)	16 (64)	8 (3)	8 (32)	* p.39
Glucocorticoids duration (days), median (IQR)	12 (3.75)	12.5 (7)	10 (6.5)	** p.022
Intravenous immunoglobulin *n* (%)	3 (12)	2 (66.6)	1 (33.3)	* p.56
Antibiotics *n* (%)	9 (37)	5 (21)	4 (16.67)	* p.285
P-ICU admission *n* (%)	3 (12)	2 (18)	1 (6.6)	* p.28
Serious neurological complication (ataxia, ADEM, GBS, epilepticus status)	4 (16)	3 (12)	1 (4)	* p.28
Antiepileptic drugs at discharge *n* (%)	3 (12)	2 (18)	1 (6)	* p.6
Length of stay in days, median (IQR)	8.5 (7.24)	9 (7)	89 (6)	** p.82

*: Fisher’s exact test; ** Mann–Whitney U test. § We included hypoxic–ischemic encephalopathy, brain tumors, one case of tuberous sclerosis, and known epilepsy at admission. ‡ Defined as any signs or symptoms of respiratory distress requiring supplementary oxygen or ventilation, as well as clinically or radiographically confirmed pneumonia. ⁋ Defined as ≥1 of the following: cloudy appeareance, white cells > 5/mm^3^ (lymphocytes), glucose < 50 mg/dL; protein > 60 mg/dL.

## Data Availability

The original contributions presented in this study are included in the article. Further inquiries can be directed to the corresponding author. The data presented in this study are not publicly available due to ethical restrictions and patient confidentiality. Requests to access the datasets should be directed to the corresponding author and will be considered on a case-by-case basis in accordance with institutional and ethical guidelines.
